# Small-Cell Lung Cancer Long-Term Survivor Patients: How to Find a Needle in a Haystack?

**DOI:** 10.3390/ijms222413508

**Published:** 2021-12-16

**Authors:** Andrea Plaja, Teresa Moran, Enric Carcereny, Maria Saigi, Ainhoa Hernández, Marc Cucurull, Marta Domènech

**Affiliations:** Catalan Institute of Oncology (ICO)-Badalona, Badalona-Applied Research Group in Oncology (B-ARGO), Medical Oncology Department, 08916 Badalona, Spain; aplaja.germanstrias@gencat.cat (A.P.); mmoran@iconcologia.net (T.M.); ecarcereny@iconcologia.net (E.C.); msaigi@iconcologia.net (M.S.); ahernandezg@iconcologia.net (A.H.); mcucurull@iconcologia.net (M.C.)

**Keywords:** small-cell lung cancer, immunotherapy, PD-L1, tumor microenvironment, molecular subtypes, predictive biomarkers, prognostic factors, long-term survivors

## Abstract

Small-cell lung cancer (SCLC) is an aggressive malignancy characterized by a rapid progression and a high resistance to treatments. Unlike other solid tumors, there has been a scarce improvement in emerging treatments and survival during the last years. A better understanding of SCLC biology has allowed for the establishment of a molecular classification based on four transcription factors, and certain therapeutic vulnerabilities have been proposed. The universal inactivation of *TP53* and *RB1*, along with the absence of mutations in known targetable oncogenes, has hampered the development of targeted therapies. On the other hand, the immunosuppressive microenvironment makes the success of immune checkpoint inhibitors (ICIs), which have achieved a modest improvement in overall survival in patients with extensive disease, difficult. Currently, atezolizumab or durvalumab, in combination with platinum–etoposide chemotherapy, is the standard of care in first-line setting. However, the magnitude of the benefit is scarce and no predictive biomarkers of response have yet been established. In this review, we describe SCLC biology and molecular classification, examine the SCLC tumor microenvironment and the challenges of predictive biomarkers of response to new treatments, and, finally, assess clinical and molecular characteristics of long-term survivor patients in order to identify possible prognostic factors and treatment vulnerabilities.

## 1. Introduction

Lung cancer is the leading cause of cancer mortality worldwide. There are two major histologic subtypes, non-small-cell lung cancer (NSCLC) and small-cell lung cancer (SCLC), which accounts for 13–15% of all lung cancers and is the sixth most common cause of cancer-related mortality [1]. SCLC is strongly associated with tobacco exposure [2], with just 2% of cases in never-smokers. Its prevalence tends to mirror the prevalence of smoking and it is decreasing in the western world. Smoking prevention constitutes the most effective measure to further decrease SCLC mortality [3].

SCLC originates from neuroendocrine cells and is characterized by a rapid growth and high metastatic potential. It commonly responds to both chemotherapy (ChT) and radiotherapy, with a response rate (RR) of around 70–80%. In spite of the initial response, it is often followed by a rapid relapse and ChT resistance, leading to a poor overall survival (OS) [4]. Around 40% of patients are diagnosed with a limited stage (LS-SCLC). Nevertheless, only 20% of patients will have long-term disease control with a median (m) OS between 15 and 20 months. The current standard of care of this subset of patients consists of concurrent chemoradiation based on four cycles of cisplatin (60–80 mg/m^2^ on day 1) and etoposide (100–120 mg/m^2^ on day 1–3) every 3 weeks, followed by prophylactic cranial irradiation (PCI) in those patients who achieve complete or partial remission [5]. However, most patients are diagnosed with extensive stage (ES-SCLC) and, due to the aggressive behavior of this disease, they have mOS between 9 and 12 months. First-line treatment consists of four to six cycles of platinum-based ChT (either cisplatin or carboplatin 5–6 AUC) and etoposide [6,7].

In the recurrence setting, a rechallenge with platinum-based ChT is recommended for patients who experience disease relapse 6 months after completion of the first-line treatment. On the other hand, for patients who relapse during the first 6 months, the use of topotecan (1.5 mg/m^2^ on day 1–5) every 3 weeks is recommended, with a RR of 7–38% and 1-year OS of 30% [8,9]. Currently, an alternative treatment is lurbinectedin, a novel anticancer agent that acts as a transcription factor inhibitor and modulates the tumor microenvironment. Lurbinectedin (3.2 mg/m^2^ on day 1) every 3 weeks has been approved by the Food and Drug Administration (FDA) after demonstrating a RR of 35% and median response duration of 5.3 months in a phase II basket trial [10]. Second-line treatment responses are higher among patients who experience longer disease control after frontline treatment [11]. After more than 25 years with no improvement in both the SCLC treatment and in survival, the emergence of immune checkpoint inhibitors (ICIs) has changed the treatment paradigm of this disease. Table 1.

First trials with immunotherapy in previously treated SCLC patients were initially published in 2017. CheckMate-032, a phase 1/2 multi-arm open-label study, assessed the safety and activity of nivolumab and different schemes of nivolumab plus ipilimumab. Ninety-eight patients received nivolumab monotherapy as third- or later-line, with a response rate (RR) of 10% (95% confidence interval (CI): 6.5–19.5%) and a median duration of response of 17.9 months. Sixty-one patients received nivolumab 1 mg/kg plus ipilimumab 3 mg/kg, with a RR of 23% and median duration of response of 7.7 months, and 54 received nivolumab 3 mg/kg plus ipilimumab 1 mg/kg, with a RR of 19% and median duration of 4 months. Programmed death-ligand 1 (PD-L1) expression was assessable in 148 (69%) of 216 patient samples, 25 (17%) had a PD-L1 ≥ 1%, and only 7 (5%) had a PD-L1 ≥ 5%. In all treatment arms, tumor responses occurred in patients irrespective of PD-L1 expression [12].

In the KEYNOTE-028, a phase Ib trial with pembrolizumab for ES-SCLC patients with a PDL1 higher than 1%, 163 patients were screened for enrolment, but only 145 had biopsy samples for PD-L1 evaluation; finally, 46 patients (31.7%) tested positive for PD-L1 expression (PD-L1 ≥ 1%) and 24 patients were treated. The objective response rate (ORR) was 33.3% and the median duration of response was 19.4 months [13]. On the other hand, the phase II trial KEYNOTE-158, also with pembrolizumab, included 107 patients independent of PDL1 expression and achieved an ORR of 18.7% [14].

Due to these results, several clinical trials were designed to test ICIs in the frame of first-line treatment for patients with naïve SCLC treatment. The phase III IMpower-133, a double-blind clinical trial, has changed the standard of care of ES-SCLC with a 2-month OS improvement by adding the PD-L1 inhibitor atezolizumab to standard ChT, which is carboplatinand etoposide, for four to six cycles, showing a mOS of 12.3 months in the atezolizumab group and 10.3 months in the placebo group (HR 0.70; 95%CI: 0.54–0.91; *p* = 0.007) [15]. A post hoc analysis studied the blood-based tumor mutational burden (bTMB) with different cut-off points and did not show a predictive value in this subset of patients. Moreover, updated OS data presented at the 2019 ESMO Congress confirmed the superior efficacy of atezolizumab plus ChT vs. ChT alone (18-month OS 34% versus 21%; *p*-value) [16].

Similar findings were reported in the CASPIAN study, a randomized open-label phase III trial, which evaluated the addition of the PD-L1 inhibitor durvalumab with or without tremelimumab to a platinum-based ChT, versus standard of care treatment combining carboplatin or cisplatin with etoposide plus prophylactic intracranial irradiation. It showed a median OS of 13.0 months in the experimental arm vs. 10.3 months in the control arm (HR 0.73, 95%CI: 0.59–0.91; *p* = 0.0047). The response rates and median time duration of response were not different between the two groups [17].

In contrast, two phase III clinical trials that also evaluated the combination of ICIs and ChT in newly diagnosed ES-SCLC patients do not show proved significant differences in OS. The phase III CA184-156, which examined the combination of ipilimumab with standard ChT platinum–etoposide, failed to prove the primary endpoint [18]. Similarly, the phase III KEYNOTE-604 analyzed the combination of pembrolizumab with platinum–etoposide, showing a longer progression-free survival (PFS) in the combination group (HR 0.75, 95%CI: 0.61–0.91, *p* = 0.0023), but no significant differences in OS [19].

Despite the survival improvement achieved with ICIs, the magnitude of this improvement remains modest in comparison with other solid tumors, such as non-small-cell lung cancer (NSCLC).

In this review, we describe the SCLC biology and molecular classification, as well as their possible therapeutic implications. We examine the SCLC tumor microenvironment (TME) and challenges of predictive biomarkers of response to new treatments. Finally, we assess clinical and molecular characteristics of long-term survivor patients in order to understand possible mechanisms involved in a better prognosis or treatment vulnerabilities.

## 2. SCLC Biology

SCLC is a high-grade neuroendocrine tumor that commonly expresses neuroendocrine markers, such as chromogranin A, synaptophysin, and neural cell adhesion molecule 1 (NCAM1) [20]. Although the genomic landscape of SCLC is complex and diverse, the functional inactivation of both *TP53* and *RB1* is essentially universal. These tumor suppressor genes play a critical role in carcinogenesis and, when defective, induce genomic instability [21].

*NOTCH*-inactivating mutations, which also act as a tumor suppressor gene in neuroendocrine tumors, are found in 25% of SCLC. Interestingly, the *NOTCH* inhibitory ligand delta-like ligand 3 (DLL3) is overexpressed in approximately 69–80% of SCLC, but not in normal tissue, making it a potential therapeutic target [22]. Even though DLL3 inhibition was thought to be a good target therapy, it failed when it was brought to a phase II clinical trial [23].

Other molecular alterations in oncogenic signaling pathways can be found in SCLC, such as mutations in cell cycle regulation genes (e.g., *Chk-1*, *Wee-1*, *CDK4/6*), alterations in receptor kinase signaling (e.g., *c-KIT*, *PI3K/AKT/mTOR*, *IGFR1*, *FGFR1* and *PTEN*), alterations in DNA repair pathways (e.g., *MYC* amplification or overexpression of PARP enzymes), and overexpression of the antiapoptotic protein BCL-2, found in 80% of SCLC [2]. The amplification of *MYC* family genes (including *MYC*, *MYCL*, and *MYCN*) occur in 20% of tumors and are mutually exclusive among them [24].

### 2.1. Molecular Subtype’s Classification

A molecular, transcriptomic, proteomic, and epigenetic study of SCLC is being deeply explored in order to assess the possible role of targeted therapy [25]. Two different molecular subtypes, which could be morphologically differentiated, were initially described: a “classical” subtype enriched by neuroendocrine biomarkers and a “variant” subtype [26].

After various attempts of the molecular classification of SCLC, four distinct subtypes defined by gene expression profiles came out, and included the following variants: the “classic”, a neuroendocrine-high or recently named the SCLC-A type; the “variant”, a neuroendocrine variable, recently named the SCLC-N type; and, finally, the dual-negative or non-neuroendocrine type, which can be divided into SCLC-Y or SCLC-I and SCLC-P [27,28,29] (Table 2).

The “classic” or SCLC-A molecular subtype is the most common subtype and represents around 40–50% of SCLC. It is the most epithelial subtype and it is characterized by the expression of transcription factor achaete-scute homologue 1 (ASCL1), which is a master regulator that induces neuronal and neuroendocrine differentiation [26]. ASCL1 targets the oncogenes *MYCL1*, *BCL2*, *RET*, *SOX2*, *INSM1*, and *NFIB*, as well as multiple genes of the *NOTCH* pathway, including *DLL3* [28].

The “variant”, also called SCLC-N, which represents around 25–30% of SCLC tumors, is characterized by the neurogenic differentiation factor 1 (NEUROD1) expression, with or without ASCL1 [30]. NEUROD1 is another neuronal master regulator of neuroendocrine differentiation and it targets *MYC* activation, as well as *INSM1* and *HES6* [4,31].

The other two subtypes, lately described, are characterized by a lack of neuroendocrine properties and a low expression of ASCL1, NEUROD1, and insulinoma-associated protein 1 (INSM1).

The non-neuroendocrine subtype SCLC-P, represents around 7–16%. It shows an overexpression of POU class 2 homeobox 3 (POU2F3) that is typically expressed in tuft cells, a rare chemosensory cell type in the pulmonary epithelium, suggesting the possibility of a distinct cell of origin [32]. Interestingly, this subtype also shows a significantly high expression of *MYC* [28]. The *MYC* oncogene family may be a key factor identifying SCLC subtypes, *MYCL1* being amplified or highly expressed in ASCL1-high tumors, and *MYC* being amplified or overexpressed in the other three subtypes [27]. *MYC* amplification has been associated with a poor prognosis and treatment resistance [33].

Finally, the SCLC-Y or I subtype represents around 15% and is characterized by a low expression of all three transcription factors, ASCL1, NEUROD1, and POU2F3. It was proposed that the overexpression of transcription factor yes-associated protein 1 (YAP-1) of the HIPPO signaling pathway could define this subtype [27]. However, Gay C et al. observed a high expression of YAP1 in both SCLC-P and SCLC-Y, proposing that this last subtype has no prevailing transcriptional signature. Furthermore, SCLC-Y has shown to be the most mesenchymal subtype. It is also named SCLC-inflamed because it expresses genes of numerous immune checkpoints and human leukocyte antigens (HLAs). Although immune cells were present in each subtype, the absolute number of T-cells, NK cells, and macrophages was markedly increased in the SCLC-Y subtype [28]. This shows that each subtype could have a different microenvironment and, therefore, different behavior and survival [34].

### 2.2. Clinical Implication of Molecular Classification

The clinical implication of this molecular classification, as of today, remains to be elucidated. Some subtype-specific vulnerabilities have been studied [35,36].

The SCLC-A subtype has shown a variable sensitivity to cisplatin. Moreover, SCLC-A models are sensitive to BCL2 inhibitors, since this subtype has the highest BCL2 protein expression [28]. However, BCL2 inhibitor trials have failed to show efficacy in unselected SCLC relapsed patients [37,38]. In the same way, the DLL3 protein is more expressed in SCLC-A tumors, suggesting that targeting DLL3 could be more effective in this subtype, but it also failed to show efficacy in a phase II trial [23,39]. LSD1 inhibition leads to *NOTCH* activation and ASCL1 suppression being an interesting target in the SCLC-A subtype [40].

SCLC-P models were the most sensitive to cisplatin, whereas SCLC-N and SCLC-Y showed platinum resistance. SCLC-N and SCLC-P have been associated with a therapeutic vulnerability to DNA damage repair and cell cycle checkpoint therapies. A robust expression of *cMYC* is a predictive biomarker for Aurora A kinase inhibitor (AURKi) sensitivity, and these subtypes are characterized by a high *MYC* expression [41,42]. A phase II trial with the AURKi alisertib in combination with paclitaxel showed efficacy signals in a relapsed or refractory population [43]. PARP inhibitors are also suspected to be more effective in these subtypes; some clinical trials with veliparib in combination with ChT have shown signals of efficacy in unselected ES-SCLC patients [44]. In addition, SCLC-P displays a unique vulnerability to IGF-1R deficiency, suggesting that IGF-1R inhibitors may constitute a target therapy in this subtype [32].

SCLC-Y displays an inflamed phenotype, enrichment of T-cells, and high expression of IFN-y response gens, suggesting that ICIs could be more effective in this subtype. An RNAseq analysis of IMpower133 subtypes demonstrated a trend toward benefit of standard ChT plus atezolizumab across all four subtypes, including a modestly improved OS in SCLC-Y [28]. On the other hand, as it is the most mesenchymal subtype, EMT reversal therapies could be an interesting treatment. Finally, this subtype is also thought to be more sensitive to mTOR, PLK, and CDK4/6 inhibitors [36].

Another interesting topic is the plasticity of the subtypes and how they change alongside treatment. It has been proposed that *NOTCH* activation is capable of mediating a switch between neuroendocrine and non-neuroendocrine SCLC [45]. Irlend et al. also observed that *MYC* drives the dynamic evolution of SCLC subtypes. *MYC* activates *NOTCH* signaling to dedifferentiate tumor cells from ASCL1+ to NEUROD1+ to the YAP1+ state. They proposed that SCLC molecular subtypes are not distinct, but a dynamic stage of tumor evolution. Thus, genetics, the cell of origin, and tumor cell plasticity determine the SCLC subtype [46]. Gay C et al. suggest that cisplatin resistance coincides with a subtype switching from SCLC-A to SCLC-Y, associated with fluctuations in *NOTCH* pathway activation [28].

## 3. SCLC Tumor Microenvironment

The immune system and TME have an important role in tumor progression, cancer phenotypes, the response to treatment, and drug resistance. An immune evasion has been correlated with a loss of neoantigens, decreased expression of major histocompatibility complex (MHC) class I or II antigens, or decreased antigen-presenting cells (APCs) [47].

Although SCLC is thought to be an immunogenic tumor due to a high tumor mutational burden (TMB) and high tumor neoantigens, the immunosuppressive stroma with lack of antigen presentation cells and limited PD-L1 expression suggest an immunologically ignorant phenotype [47,48]. Therefore, it is known that SCLC displays an immunosuppressive phenotype with a low infiltration by immune cells, low level of tumor-infiltrating lymphocytes (TILs), low ratio of T effector to T regulator, and the presence of immature myeloid cells [49,50]. Moreover, some mechanisms of immune checkpoint inhibitor resistance present in SCLC include: a low expression of HLAs, interferon signatures and low expression of PDL1, and the T-cell immunoglobulin domain, mucin domain 3 (TIM3), and lymphocyte activation gene 3 (LAG3) [51], as well as the upregulation of CD47 [52].

However, data on the immunophenotype of SCLC is very limited, probably due to the difficulty of obtaining proper tumor samples as a consequence of the rare surgical resection of this disease and the limited access to biopsies due to their central localization. Furthermore, biological samples often have scarce cellularity, with a high percentage of necrosis; thus, diagnosis is based on cytological analysis.

### 3.1. Predictive Biomarkers of Response to ICIs

The arrival of immunotherapy has forced the need to look for new predictive biomarkers of response (Table 3). The most deeply studied biomarkers are TMB and PD-L1 expression [53,54]. Tumor-based TMB (tbTMB) is thought to have a potential predictive value, whereas PD-L1 expression and blood-based TMB (bTMB) have not shown a robust predictive impact related to the immunotherapy benefit [55].

#### 3.1.1. TMB

TMB is defined as the total number of non-synonymous mutations within a tumor genome. SCLC has a high median TMB—around eight mutations per megabase—which is predictive of a larger number of tumor neoantigens and has shown to be a biomarker of response to ICIs in some tumors, such as melanoma [56,57]. However, the predictive value of TMB is controversial in SCLC.

The use of tbTMB showed early promise as a predictor of the benefit to ICIs in patients with relapsed SCLC. In a pooled exploratory analysis from the CheckMate-032 trial, the tbTMB was assessed using whole-exome sequencing (WES), and tertiles were defined as <143 mutations (low), 143–247 mutations (intermediate), and ≥248 mutations (high). The tbTMB was evaluable in 212 (53%) patients who received nivolumab with or without ipilimumab. In those patients, a high tbTMB demonstrates a better ORR, PFS, and OS compared with a low/medium tbTMB. ORRs with nivolumab in monotherapy were 5%, 7%, and 21% in the low, intermediate, and high tbTMB tertiles, respectively, whereas the median OS was 3.1, 3.9, and 5.4 months (*p*-value), respectively. The combination of nivolumab 1 mg/kg and ipilimumab 3 mg/kg revealed similar findings, with an ORR of 22%, 16%, and 46% and a mOS of 3.4, 3.6, and 22 months (*p*-value), respectively [12,58].

KEYNOTE-028, also explored tbTMB by WES, showing a statistically significant correlation between the tbTMB and ORR and median PFS. However, due to the small sample analyzed, no conclusions can be drawn [13]. The tbTMB does not seem to correlate with PDL1 expression in any of these studies [59].

On the other hand, the bTMB has not shown to have a predictive value of the immunotherapy response in patients receiving ChT plus ICI in the first-line setting. The IMpower-133 study assessed the bTMB using cfDNA sequenced and next generation sequencing (NGS) of 394 cancer-associated genes. The bTMB could be determined in 351 (87%) patients and grouped according to two pre-specified arbitrary cut-offs of 10 and 16 mutations/ Mb. Given the similar levels of benefit in OS across bTMB subgroups, this biomarker is not thought to be a good predictor of response [15]. Similarly, the tbTMB was assessed in 35% of the population of the CASPIAN trial, with no predictive role for OS [17].

Although prospective studies are needed, these studies suggest that the tbTMB may have a predictive role toward the response to ICI in patients with relapsed SCLC, whereas the tbTMB does not seem to have a predictive OS value in patients receiving ICIs in combination with ChT as first-line treatment. Further studies are needed to determine the best source of assessing the TMB, as well as to establish the appropriate cut-offs [56].

#### 3.1.2. PD-L1

SCLC is characterized by a low PD-L1 expression compared to NSCLC. PD-L1 expression in >1% of tumor cells is present in only a minority (~20%) of SCLC specimens [12,60]. Moreover, the majority of PD-L1 expression is found on the immune cells instead of tumor cells (50 vs. 6%, respectively) [61].

Some retrospective studies showed that the expression of PD-L1 was significantly higher in early-stage SCLC than in extended disease (*p* = 0.011), and resulted in an independent prognostic biomarker of a favorable outcome [62,63,64]. In contrast, some studies of early stages of SCLC suggest that higher expressions of PDL-1 confer a worse prognostic in SCLC resected and that higher PDL1 expression is associated with a higher stage disease [65].

The expression of PD-L1 on tumor and immune cells was firstly evaluated as a predictive biomarker of response to ICIs in second-line or later. In CheckMate-032, the tumor PD-L1 expression was evaluated as the tumor proportion score (TPS) or the percentage of tumor cells staining positive by immunohistochemistry (IHC). One hundred and forty-eight (69%) samples were obtained within 3 months of beginning ICI and no significant association between PDL1 expression and the ORR was found, neither with nivolumab monotherapy nor with the combination of nivolumab 1 mg/kg and ipilimumab 3 mg/kg [12].

In contrast, KEYNOTE studies analyzed PD-L1 using a combined positive score (CPS), which is the number of PD-L1 staining cells (tumor cells, lymphocytes, macrophages) divided by the total number of viable tumor cells. These studies observed that PD-L1 positivity did correlate with the benefit from pembrolizumab. In KEYNOTE-028, a CPS of ≥1% was an inclusion criteria, and patients showed an ORR of 33% [13]. KEYNOTE-158 stratified patients into two arms: CPS ≥ 1% and CPS < 1%. The ORRs were 35.7% vs. 6%, and the mOS was 14.6 vs. 7.7 months, respectively [14]. Even though these results could suggest that PDL1-low tumors do not respond to ICIs, the low level of PD-L1 expression and the small number of patients included do not allow us to confirm this predictive value [66]. An exploratory analysis of Gadgeel et al. showed that, despite the low prevalence of PD-L1 expression on tumor cells, its expression on stroma may be more frequent and a possible predictive biomarker of the benefit from ICI [67].

PD-L1 expression has also been evaluated in the first-line setting. Of the 403 patients enrolled in the IMpower-133 trial, 137 had evaluable tumor material and 68 (49.6%) had <1% PD-L1 expression on immune cells. No significant improvement in OS was detected among those with PD-L1 expression on tumor cells or immune cells ≥ 1% or ≥5%. In patients with both tumor and immune cell PD-L1 expression < 1%, a statistically significant improvement in the OS was observed in those receiving ChT plus atezolizumab vs. ChT plus placebo (median OS 10.2 months versus 8.3 months, respectively; HR 0.51, 95%CI: 0.30–0.89) [16]. Similarly, in the CASPIAN trial, the PDL-1 expression was evaluable in 52% of patients. Ninety-five and 78% of patients had a PD-L1 expression < 1% in tumor and immune cells, respectively. The combination of ChT and ICIs was associated with improved OS regardless of PD-L1 expression [17]. Finally, in the KEYNOTE 604 trial, the CPS PD-L1 expression was not a predictive biomarker of PFS or OS [19]. These inconsistent findings suggest that PD-L1 expression is not a strong predictive biomarker of OS in patients with SCLC receiving ChT in combination with an ICI.

Collectively, PD-L1 has several limitations in SCLC, and the method of evaluating PD-L1 is still unknown.

#### 3.1.3. Other Emerging Biomarkers

As is well known in NSCLC, a high tumor infiltration of T lymphocytes is a predictive marker of the benefit to immunotherapy. Nonetheless, immunosuppressive cells also have a role in the TME of solid tumors. FOXP3+ T lymphocytes are regulatory T-cells with a controversial prognostic role. In SCLC, a high tumor infiltration by FOXP3+ T lymphocytes has been associated with metastatic patients and a lower survival [68]. In contrast, in another retrospective study, the expression by the immunohistochemistry of FOXP3-positive TILs, which classifies immunosuppressive lymphocytes, showed a positive prognostic impact in stage I-III SCLCs [69]. In the same way, another retrospective study included 102 patients diagnosed with stage I to III SCLC and found that the FOXP3 level was associated with a higher recurrence-free survival. In addition, the expression of FOXP3 was also associated with the PD1, CD4, CD8, and CD3 status [70]. The modulation of T reg lymphocytes could be an interesting strategy related to immunomodulation in these types of tumors [66].

Cytokines are small molecules that have an important role in cell signaling and immunomodulation; it is known that the homeostasis between the several cytokines is disrupted in TME. A study of SCLC patients treated with ChT and ipilimumab showed an increase in IL-2 levels in those patients with better OS. On the contrary, higher levels of IL-6 and TGF-B were associated with resistance to ipilimumab. Even though the data reported are related to ipilimumab treatment, it has been proposed that baseline levels and changes in the level of cytokines could be a predictive biomarker of response to immunotherapy [71].

In the 1980s, a deficiency of MHC1 antigens (HLA-A, B, C and β2-microglobulin]) in the SLCL human cell line in comparison with NSCLC was already described [72]. A low expression of MHC has been associated with chemoresistance. In addition, the lack of MHC class I molecules is thought to be the main mechanism of the immune evasion of SCLC; thus, the tumor neoantigens cannot be presented to CD8+ T-cells, conferring a less immunogenic TME than NSCLC [73,74].

Muppa et al. demonstrated that higher concentrations of monocytes, immune-suppressive lymphocytes, and macrophages were present in long-term survivors (OS > 4 years), suggesting that these cells could be a predictive biomarker of good prognosis. Macrophages act as antigen presentation cells, and a positive association between macrophage infiltration and the tumor stage has been found [75]. Moreover, SCLC shows a widespread CD47 expression that inhibits SIRPα, a molecule that leads the macrophage activation. Thus, CD47 could be an immunotherapeutic target in SCLC. In preclinical studies, CD47 ablation was effective in inhibiting tumor growth in both immunocompromised and immunocompetent models [47,76].

Another possible biomarker could be B7-H6 (NCR3LG1), which is a novel ligand from the B7 family that is not present in human normal tissues, but is only present in tumor tissues. It has been found to be associated with a longer PFS and higher immune infiltration. B7-H6 binds to NKp30 enhancing antitumor NK cell cytotoxicity, but, paradoxically, a negative correlation with activated NK cells was observed [77].

## 4. Long-Term Survivors in SCLC

Despite the high lethality of SCLC, both in localized and extensive stages, some patients have shown survival times that exceed 24 months, and are considered long-term survivors [78]. Some studies have analyzed the clinical characteristics and biologic characteristics of these patients to identify possible prognostic factors.

### 4.1. Clinical Characteristics of Long-Term Survivors

Several prognostic factors have been studied in the last 40 years, the disease stage and performance status being the most consistent [79,80]. Other clinical prognostic factors have been shown to have an impact in retrospective studies, such as weight loss, age < 60 years, male gender, the presence of brain and lymph node metastases, baseline lymphopenia, a high ratio of neutrophil-to-lymphocyte, a high ratio of platelet-to-lymphocyte [81], a high LDH [82], and a lack of response to cisplatin-based ChT, thoracic radiotherapy, or PCI [83].

SCLC has also been associated with an increased incidence of paraneoplastic syndromes. It has not been associated with long-term survivors; nonetheless, the presence of paraneoplastic antibodies has been correlated with better prognosis [84].

Lewinski et al. studied a cohort of 111 long-term survivor patients defined as OS > 24 months; in this study, 79.2% had a LD-SCLC. A total of 45.9% of the patients were re-treated for relapse before 24 months, and 41% died between the second and third years. However, after the third year, the mortality decreased substantially without any relapse beyond 5 years. The development of a second primary tumor, mainly lung cancer, was the major cause of death after 5 years. They concluded that 3-year survival allows for predicting a significantly reduced risk of recurrence [85].

Jacoulet et al. also analyzed a cohort of 155 long-term survivor patients, defined as OS > 30 months, of whom, 87% had a LD-SCLC. From 43 relapsing patients, 76.7% benefited from a second-line treatment, with a median survival since retreatment of 12 months, and 5-year and 10-year survival rates of 68% and 44%, respectively. The risk of relapse became less than 10% beyond 5 years and 13% of patients developed a second primary cancer in a mean time of 58.6 months. An age > 60 years at the time of diagnosis was found to be an independent factor increasing the risk of relapse beyond 30 months [86].

### 4.2. Biological Characteristics of Long-Term Survivors

TME has also been studied in SCLC long-term survivors, as immune cell infiltration has shown to be a predictive biomarker in several solid tumors. Several retrospective studies have shown that the presence of TIL is a good prognostic marker in different cohorts of SCLC patients with non-homogeneous characteristics [68]. Muppa et al. observed that long-term survivors have an increased number of TILs, as well as a less tumor-suppressive microenvironment, with a low ratio of suppressive immune cells to CD3-positivelymphocytes [75]. Moreover, TILs were studied in resected SCLC brain metastases, and the greater the presence of CD45RO+ TILs, the longer the median survival (mOS was 11 months in patients with CD45RO+ TIL vs. 5 months in patients without CD45RO+ TIL) [87]. Koyama et al. demonstrated that long-term survivors maintain a high T-effector-to-T-regulator-cells ratio, whereas patients with recurrent disease show a low ratio [88].

In summary, TME results in being dynamic during tumor evolution. A higher tumor cell infiltration is related to early stages and a better prognosis in SCLC, regarding the limitation that samples are heterogeneous. Further studies are needed to identify the best approach to assess these biomarkers and their implications on the microenvironment modulation [56].

In the last years, new technologies have been developed in order to have a better understanding of cellular heterogeneity and the cell–cell interaction between the tumor and tumor microenvironment [89]. Single-cell RNA-sequencing (scRNA-seq) has improved our knowledge on cell functional heterogeneity, but, as it requires a dissociation of the tissue of interest, it has a lack of spatial information. Thus, a spatial transcriptomic has emerged that allows for a better understanding of tissue functional organization and cell interaction in their environment by preserving the tissue architecture [90]. Differential defects in HLA molecules have been studied using spatial techniques and have been associated with a distinct tumor microenvironment and survival in NSCLC patients [91]. These revolutionary techniques could help in the understanding of the heterogeneity of SCLC phenotypes, their interaction with TME, and dynamic evolution.

## 5. Future Perspectives and Conclusions

SCLC is an aggressive and lethal disease characterized by an acquired chemoradiotherapy resistance, extraordinary metastatic potential, and poor overall survival in both limited and extended diseases. After decades without progress in treatment, the addition of ICIs to platinum-based first-line ChT has resulted in a modest change in survival. However, unlike other tumors, the immunotherapy benefit is scarce and no predictive biomarkers of response have been established yet.

SCLC was considered to be an immunogenic tumor with high somatic mutation rates due to tobacco exposure, and, therefore, was predicted to induce strong T-cell responses. However, SCLC also has an immunosuppressive TME, characterized by high regulatory T-cells and a low expression of MHC class I molecules. TME plays a critical role in the tumor prognosis and response to treatments and may explain the conflicting efficacy of ICIs in SCLC. The beneficial effect of ICIs that enhance the activity of the T-cell by blocking CTLA4, PD1, and PDL1 is limited to around 15% of SCLC patients [92]. A deep exploration should be carried out in order to identify more adequate immune modulations or combinations in order to increase the benefit of these therapies. Current trials are investigating combinations, with alternative ICIs targeting LAG-3, TIM-3, and TIGIT. Small molecular inhibitor combinations are also being studied, such as the CDK4/6 inhibitor or PARP inhibitors. Additional immunomodulatory pathways that affect the innate immunity to induce antitumor activity offer new strategies, such as a TLR7 or TLR9 agonist and natural killer cell activators, among others [93]. Finally, epigenetically targeted agents, cellular therapy, and vaccines that can be used to induce immune responsiveness are also being explored. Some potential targets of chimeric antigen receptor T-cell (CART) therapy are CD56, DLL3, and CD47 cell surface molecules, which are highly expressed in SCLC [94].

**Figure 1:**
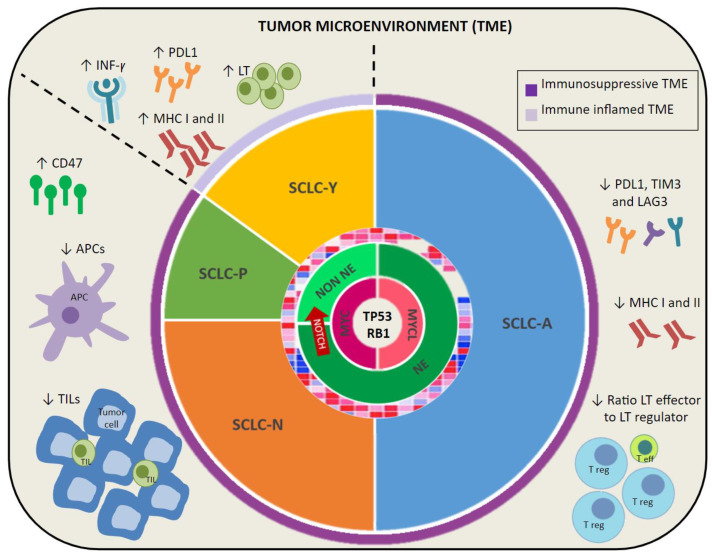
SCLC molecular subtypes and tumor microenvironment (TME). This figure shows SCLC genetic alterations with a universal mutation of *RB1* and *TP53*, along with mutations on *MYC* family and *NOTCH* that could guide a plasticity between neuroendocrine and non-neuroendocrine subtypes. Molecular classification based on four transcriptional factors is also shown: SCLC-A (ASCL1), SCLC-N (NEUROD1), SCLC-P (POU2F3), and SCLC-Y (YAP1). Finally, an immunosuppressive TME is represented, consisting of a low expression of PDL1, LAG3, and TIM3, MHC class I and II, APCs, TILs, low ratio lymphocyte T effector to regulator, and upregulation of CD47. However, SCLC-Y subtype is characterized by an immune inflamed TME with higher expression of PDL1, MHC molecules, immune cell infiltration, and INF-y signaling. PD-L1: programmed cell death ligand 1. TIM-3: T-cell immunoglobulin domain and mucin domain 3. LAG-3: lymphocyte activation gene 3. APCs: antigen presentation cell. TILs: tumor-infiltrating lymphocytes. MHC: major histocompatibility complex. LT eff: lymphocyte T effector. LT reg: lymphocyte T regulator. NE: neuroendocrine. NON NE: non-neuroendocrine.

Although there has been extensive research to determine the molecular characteristics of SCLC, no targeted treatment has changed the treatment paradigm of this disease. The genetic homogeneity of SCLC, with a universal inactivation of two key suppressor genes, *TP53* and *RB1*, has led to it being considered as a single entity. However, the improved understanding of SCLC biology has proven an inter- and intra-tumoral heterogeneity, and a molecular characterization based on the differential expression of key transcription regulators has been proposed. This phenotypical classification could suppose an initial rationale on treatment the vulnerabilities of each subtype. Nevertheless, the plasticity between subtypes during treatment mediated by *NOTCH* and *MYC* mutation can determine the evolution to drug resistance. Therefore, it would be interesting to determine the molecular subtype and, especially, to monitor the dynamic changes alongside treatment, and, thus, to guide a more targeted treatment (Figure 1).

Lastly, long-term survivor patients have been studied in order to identify clinical and biological prognostic factors in SCLC. The disease stage and performance status are the most consistent clinical good prognostic factors. Notably, a reduced risk of recurrence after 3 years and a high risk of developing a second primary cancer have been found in these patients. On the other hand, some of the biological good prognostic factors found in long-term survivors are a less immunosuppressive microenvironment, with a greater immune cell infiltration, higher number of TILs, MHC molecules, and PDL1, and a higher T-effector–T-regulator ratio.

In conclusion, SCLC is a complex disease with high intra and intertumoral heterogeneity and a rapid development of treatment resistance. It would be interesting to study molecular subtypes and TME throughout the disease in order to better understand its mechanisms of resistance and guide a target therapy to improve outcomes.

## Figures and Tables

**Table 1:** Key clinical trials of immune checkpoint inhibitors in extensive stage of small-cell lung cancer (ES-SCLC).

Trial	Phase	Treatment Arms	Patients	ORR (%)	PFS (Months)	OS (Months)
Second line and beyond						
CheckMate 032 (2016) [12]	I/II	Nivolumab 3 mg/kg v. Nivolumab 1 mg/kg + Ipilimumab 3 mg/kg vs. Nivolumab 3 mg/kg + Ipilimumab 1 mg/kg	216	10 vs. 23 vs. 19	1.4 vs. 2.6 vs. 1.4	4.4 vs. 7.7 vs. 6.0
KEYNOTE 028 (2017) [13]	IB	Pembrolizumab	24	33.3	1.9	9.7
KEYNOTE 158 (2018) [14]	II	Pembrolizumab	107	18.7	2.0	9.1
First line						
IMpower 133 (2018) [15,16]	III	Atezolizumab + carboplatin + etoposide vs. placebo + carboplatin + etoposide; maintained with atezolizumab vs. placebo	403	60.2 vs. 64.4	5.2 vs. 4.3 (HR: 0.77; 95%CI: 0.62–0.96; *p* = 0.02 *)	12.3 vs. 10.3 (HR: 0.70; 95%CI: 0.54–0.91; *p* = 0.007 *)
CASPIAN (2019) [17]	III	Durvalumab ± tremelimumab + platinum-etoposide vs. platinum-etoposide; maintained with durvalumab	805	79.5 vs. 70.3	5.1 vs. 5.4 (HR: 0.78; 95%CI: 0.65–0.94)	13.0 vs. 10.3 (HR: 0.73; 95%CI: 0.59–0.91; *p* = 0.0047 *)
CA184–156 (2016) [18]	III	Ipilimumab + platinum-etoposide vs. platinum-etoposide + placebo; maintained with ipilimumab vs. placebo	1132	62 vs. 62	4.6 vs. 4.4 (HR: 0.85; 95%CI: 0.75–0.97, *p* = 0.016)	11.0 vs. 10.9 (HR: 0.94; 95%CI: 0.81–1.09, *p* = 0.3775)
KEYNOTE-604 (2018) [19]	III	Pembrolizumab + platinum-etoposide vs. placebo + platinum-etoposide; maintained with pembrolizumab vs. placebo	453	70.6 vs. 61.8	4.5 vs. 4.3 (HR: 0.75; 95%CI: 0.61–0.91; *p* = 0.0023 *)	10.8 vs. 9.7 (HR: 0.80; 95%CI: 0.64–0.98; *p* = 0.0164 ^†^)

ORR: objective response rate, PFS: progression-free survival, OS: overall survival, HR: hazard ratio, CI: confidence interval. * significant results. ^†^ significance threshold was not met.

**Table 2:** SCLC molecular classification: nomenclature evolution along years and characteristics.

Year	Neuroendocrine		Non-Neuroendocrine	
1985 [26]	Classic	Variant		
2013 [30]	ASCL1-high	NEUROD1-high		
2915 [21]	SC-E2	SC-E1		
2016 [31]	ASCL1-high	NEUROD1-high	Double negative	
2017 [29]	INSM1			YAP1
2018 [32]			POU2F3	
2019 [27]	SCLC-A	SCLC-N	SCLC-P	SCLC-Y
2021 [28]	SCLC-A	SCLC-N	SCLC-P	SCLC-I
Molecular subtype characteristics				
Proportion	40–50%	25–30%	7–16%	15%
Targets	*MYCL1*, *BCL2*, *RET*, *SOX2*, *INSM1*, *NFIB*, *NOTCH*, *DLL3*	*MYC*, *INSM1*, *HES6*	*MYC*, *IGF1R*	*mTOR*, *PDL1*, *CDK4/6*, *IFNy*
Potential targeted therapy	BCL2 inhibitors DLL3 inhibitors LSD1 inhibitors	AURKA inhibitors PARP inhibitors	IGFR1 inhibitors AURKA inhibitors PARP inhibitors	ICIs mTOR inhibitors CDK4/6 inhibitors EMT reversal therapies

ASCL1, achaete-scute homologue 1; NeuroD1, neurogenic differentiation factor 1; POU2F3, POU class 2 homeobox 3; YAP1, yes-associated protein 1; INSM1, insulinoma-associated protein 1; BCL2, B-cell lymphoma 2; DLL3, delta-like ligand 3; LSD1, lysine-specific histone demethylase 1; PARP, poly (ADP-ribose) polymerase; AURKA/B, Aurora kinase A/B; IGF-R1, insulin-like growth factor 1 receptor; ICIs, immuno checkpoint inhibitors; mTOR, mammalian target of rapamycin; CDK4/6, cyclin-dependent kinase 4/6; EMT, epithelial–mesenchymal transition.

**Table 3:** Predictive biomarkers of response to immune checkpoint inhibitors in clinical trials.

Biomarker	ORR (%)	PFS (Months)	OS (Months)
Second line and beyond			
PDL1 TPS [12]	Not predictive value: similar benefit regardless PDL1		
PDL1 CPS [13,14]	ORR 33 in CPS > 1 35.7 vs. 6 in CPS ≥ 1 vs. <1	2.1 vs. 1.9 in CPS ≥ 1 vs. <1	14.6 vs. 7.7 in CPS ≥ 1 vs. <1
TMB [12]	21.3 vs. 6.8 vs. 4.8 in patients receiving nivolumab with high, medium, and low TMB tertile, respectively.	1.3 vs. 1.3 vs. 1.4 in patients receiving nivolumab with high, medium, and low TMB tertile, respectively.	3.1 vs. 3.9 vs. 5.4 in patients receiving nivolumab with high, medium, and low TMB tertile, respectively.
	46.2 vs. 16 vs. 22.2 in patients receiving nivolumab + ipilimumab with high, medium, and low TMB tertile.	1.5 vs. 1.3 vs. 7.8 in patients receiving nivolumab + ipilimumab with high, medium, and low TMB tertile, respectively.	3.4 vs. 3.6 vs. 22 in patients receiving nivolumab + ipilimumab with high, medium, and low TMB tertile, respectively.
First line			
Tumor or immune PDL1 expression [15,17]			Not predictive value among patients with PDL1 ≥ 1% or ≥5%. Patients with PD-L1 < 1% derived the highest level of benefit from addition of atezolizumab to chemotherapy (HR 0.51).
			Similar benefit regardless PDL1 from addition to durvalumab.
PDL1 CPS [19]		Not predictive value: similar benefit from addition to pembrolizumab regardless PDL1.	Not predictive value: similar benefit from addition to pembrolizumab regardless PDL1.
TMB [17]			Not predictive value: similar benefit from addition of durvalumab irrespective of TMB.
Blood-based TMB [15]			Not predictive value: similar benefit from addition of atezolizumab: ≥10 mut/Mb HR 0.70; >10 mut/Mb HR 0.68; <16 mut/Mb HR 0.71; ≥16 mut/Mb HR 0.63

ORR: objective response rate, PFS: progression-free survival, OS: overall survival, TPS: tumor proportion score, CPS: combined positive score, TMB: tumor mutational burden: HR: hazard ratio, mut/Mb: mutations per megabase, PD-L1: programmed cell death ligand 1.

## Data Availability

Data sharing not applicable.
